# Chronic kidney disease is associated with a risk of higher mortality following total knee arthroplasty in diabetic patients: a nationwide population-based study

**DOI:** 10.18632/oncotarget.22215

**Published:** 2017-10-31

**Authors:** Liang-Tseng Kuo, Su-Ju Lin, Chi-Lung Chen, Pei-An Yu, Wei-Hsiu Hsu, Tien-Hsing Chen

**Affiliations:** ^1^ Division of Sports Medicine, Department of Orthopaedic Surgery, Chang Gung Memorial Hospital, Chiayi, Taiwan; ^2^ Chang Gung University of Science and Technology, Chiayi, Taiwan; ^3^ Division of Nephrology, Department of Medicine, Chang Gung Memorial Hospital, Chiayi, Taiwan; ^4^ College of Medicine, Chang Gung University, Taoyuan, Taiwan; ^5^ Division of Cardiology, Department of Medicine, Chang Gung Memorial Hospital, Keelung, Taiwan

**Keywords:** total knee arthroplasty, diabetes mellitus, chronic kidney disease, mortality, periprosthetic joint infection

## Abstract

Diabetes and chronic kidney disease (CKD) are associated with a higher rate of complications in patients undergoing total knee arthroplasty (TKA). The purpose of this study was to determine the effects of CKD and diabetes in patients after TKA. Diabetic patients who received unilateral primary TKA between January 2008 and December 2011 were enrolled. The follow-up period was more than 6 months. The primary outcome was a TKA-related infection and the secondary outcome was all-cause mortality. The study cohort included 13844 patients who were followed for a mean period of 2 years, of whom 1459 (10.5%) had CKD. The patients with CKD were older than those without CKD (71.6 versus 70.3 years, *P*<0.0001) and had higher rates of hypertension, gouty arthritis, ischemic heart disease, chronic pulmonary obstructive disease, pulmonary embolism and deep vein thrombosis (all *P*<0.0001). After adjustment of comorbidities, the CKD group had a higher incidence of urinary tract infections (OR: 1.61, 95% CI: 1.19-2.17). There were no significant differences in wound infections, pneumonia, pulmonary embolism or in-hospital death between the two groups. After adjustment of confounders, the CKD group had higher rates of myocardial infarction (HR: 2.06, 95% CI: 1.26–3.39) and mortality (HR: 1.99, 95% CI: 1.59–2.48). The risk of TKA-related infection during follow-up was comparable between the two groups (HR: 1.31, 95% CI: 0.94–1.82). In conclusion, CKD is associated with increased risks of urinary tract infections, myocardial infarction and all-cause mortality after TKA. Surgeons should be aware of this when evaluating TKA patients with renal disease.

## INTRODUCTION

Periprosthetic joint infection (PJI) is a serious problem following total knee arthroplasty (TKA) [1, 2]. The associated risk factors include surgical, environmental and host factors [3]. Orthopaedic surgeons have made great efforts to improve surgical outcomes, however medical comorbidities such as diabetes mellitus (DM) and chronic kidney disease (CKD) are still common risk factors leading to PJI [2, 4, 5].

It has been reported that patients with DM have more perioperative complications after TKA compared with patients without DM [6, 7]. In addition, CKD has an impact on a patient’s immunity, resulting in poorer outcomes following orthopaedic procedures [5, 8–11]. Furthermore, patients with end-stage renal disease have been reported to have a higher incidence of complications including infections after TKA [12–14]. However, no comparative study has investigated the effect of renal disease on the incidence of PJIs and associated outcomes following TKA in patients with DM.

The objectives of this study were to compare the incidence of complications and mortality following TKA in diabetic patients with and without nephropathy. We hypothesized that diabetic patients with nephropathy would have higher rates of complications such as PJI and poor surgical outcomes than those without nephropathy.

## RESULTS

### Patient’s characteristics

**Figure 1:**
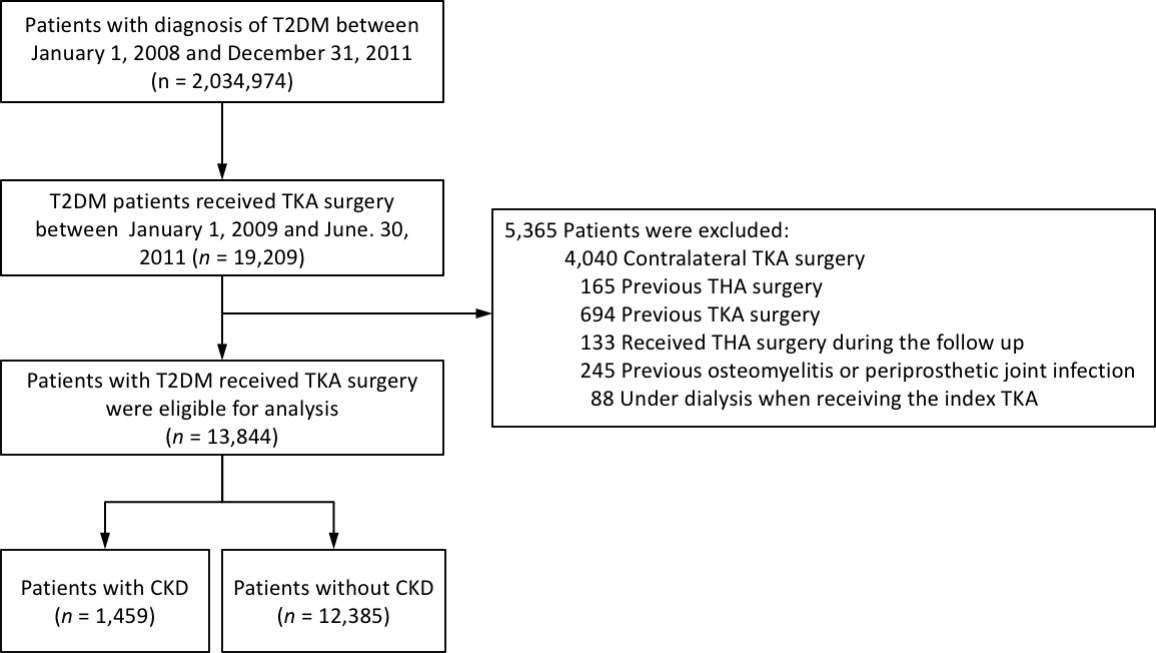
The enrollment of the study subjects

Of the 13844 diabetic patients with primary TKA, 1459 had CKD (CKD group) and the remaining 12385 patents did not (non-CKD group) (Figure 1). As shown in Table 1 , the CKD group was 1.3 years older than the non-CKD group, and the CKD group had higher rates of hypertension, gouty arthritis, cirrhosis, ischemic heart disease, chronic obstructive pulmonary disease, malignancy, pulmonary embolism and a history of deep vein thrombosis (all *P*<0.05) (Table 1).

**Table 1:** Characteristics of the study cohort

Variable	CKD (*n*=1459)	Non-CKD (*n*=12385)	*P* value
Age, Mean (*SD*)	71.6 (7.6)	70.3 (7.6)	<0.0001*
Age group			
< 65 years	241 (16.5)	2466 (19.9)	<0.0001*
65-74 years	718 (49.2)	6386 (51.6)	
≥ 75 years	500 (34.3)	3533 (28.5)	
Gender			
Male	477 (32.7)	2738 (22.1)	<0.0001*
Female	982 (67.3)	9647 (77.9)	<0.0001*
Comorbidity			
Hypertension	1305 (89.4)	10140 (81.9)	<0.0001*
Dyslipidemia	611 (41.9)	5159 (41.7)	0.8703
Gouty arthritis	366 (25.1)	1262 (10.2)	<0.0001*
Cirrhosis	31 (2.1)	165 (1.3)	0.0154*
Ischemic heart disease	421 (28.9)	2740 (22.1)	<0.0001*
Rheumatoid arthritis	50 (3.4)	380 (3.1)	0.4550
COPD	128 (8.8)	685 (5.5)	<0.0001*
Immune disease	67 (4.6)	466 (3.8)	0.1193
Malignancy	99 (6.8)	552 (4.5)	<0.0001*
Pulmonary embolism	5 (0.3)	4 (0.0)	<0.0001*
Deep vein thrombosis	15 (1.0)	51 (0.4)	0.0012*
Cerebral vascular accident	145 (9.9)	1046 (8.5)	0.0545
Follow up (years)	1.9 (1.0)	2.1 (1.0)	<0.0001*

CKD = chronic kidney disease; COPD = chronic obstructive pulmonary disease; SD = standard deviation.

* *P* value < 0.05.

### In-hospital complications

As shown in Table 2, the CKD group had a higher risk of developing urinary tract infections during the index admission than the non-CKD group (odds ratio [OR], 1.61 95% CI, 1.19-2.17, *P*=0.0020). There were no significant differences in other complications including pneumonia, acute PJI, or in-hospital death between the two groups.

**Table 2:** In-hospital complications

Variable	CKD (*n* = 1459)	Non-CKD (*n* = 12385)	CKD vs. Non-CKD OR (95% CI)^a^	*P* value
Pneumonia	4 (0.3)	28 (0.2)	0.91 (0.31–2.68)	0.8670
Urinary tract infection	54 (3.7)	310 (2.5)	1.61 (1.19–2.17)	0.0020*
Acute PJI	5 (0.3)	60 (0.5)	0.73 (0.29–1.83)	0.4979
In hospital death	2 (0.1)	7 (0.1)	1.53 (0.30–7.74)	0.6075

PJI = periprosthetic joint infection; OR = odds ratio; CI = confidence interval.

^a^ Variables listed in Table 1 (except for follow up year) were adjusted.

* *P* value < 0.05.

### Outcomes after discharge of the index admission

**Figure 2:**
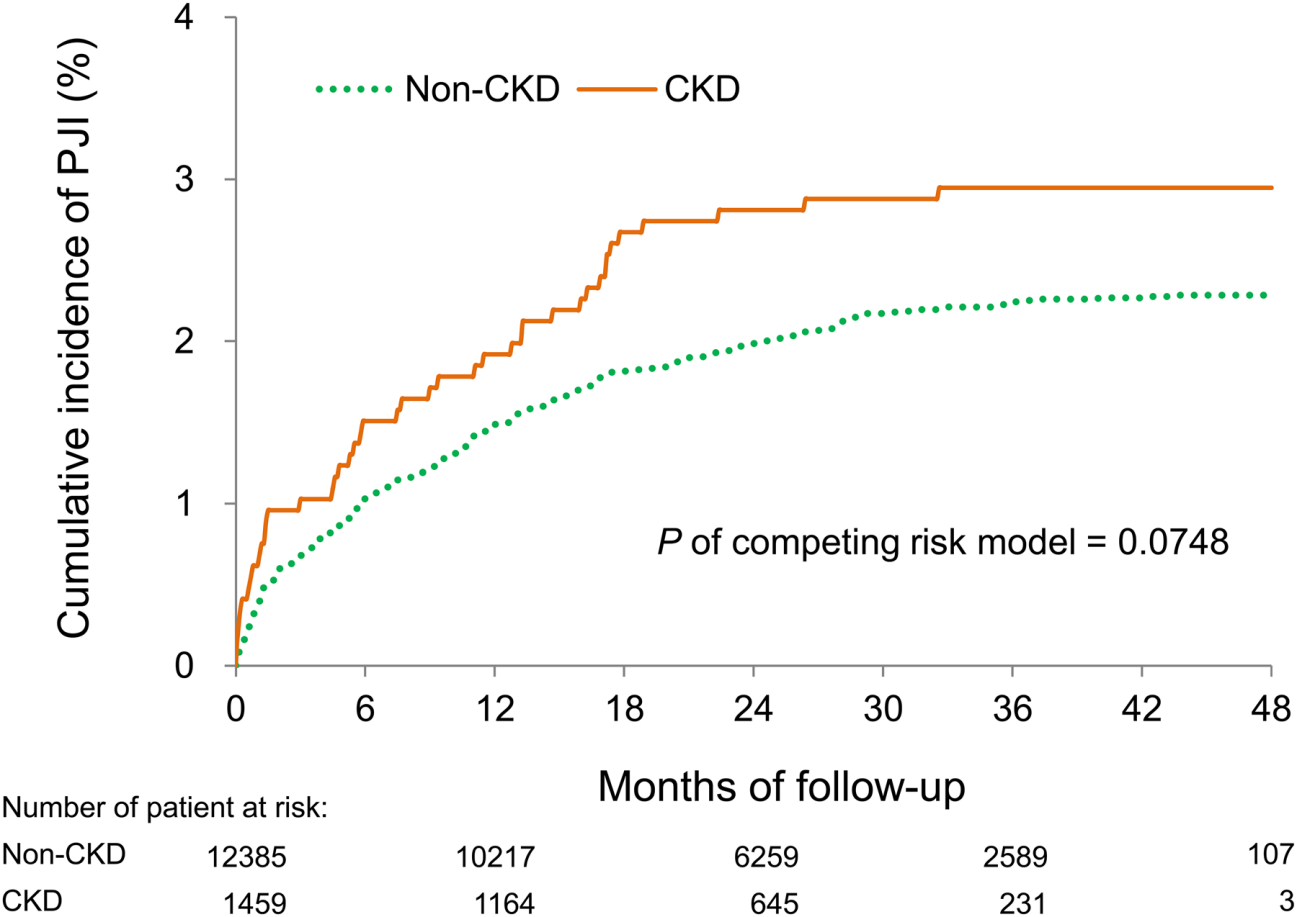
Cumulative incidence of PJI Patients with CKD tended to have a higher cumulative incidence of PJI than those without CKD, although the difference was not statistically significant (*P*=0.0748, competing risk survival model).

Table 3 shows the results of late complications including infection outcomes, cardiovascular events, re-admission and all-cause mortality. With regards to infection outcomes, the CKD group had a trend of being prone to developing PJIs compared to the non-CKD group (hazard ratio [HR]: 1.31; 95% CI, 0.94–1.82; *P*=0.1133) after adjusting for possible confounding variables. With regards to cardiovascular events, the CKD group had a higher risk of acute myocardial infarction and major adverse cardiac events than the non-CKD group (*P*<0.05). The cumulative incidence of PJI was slighter higher in the CKD group, although the difference was not statistically significant (*P*=0.0748, competing risk survival model) (Figure 2).

**Table 3:** Outcomes after discharge of the index admission

Variables	CKD (*n* = 1459)	Non-CKD (*n* = 12385)	CKD vs. Non-CKD HR (95% CI)^b^	*P* value
**Infection outcome**				
Superficial wound infection	14 (1.0)	93 (0.8)	1.27 (0.71–2.26)	0.4252
PJI requiring debridement	30 (2.1)	210 (1.7)	1.18 (0.77–1.80)	0.4397
PJI requiring implants removal	8 (0.6)	35 (0.3)	1.76 (0.80–3.85)	0.1601
Any PJI	43 (3.0)	283 (2.3)	1.31 (0.94–1.82)	0.1133
**Cardiac outcome**				
Pulmonary embolism	2 (0.1)	34 (0.3)	0.43 (0.10–1.80)	0.2479
Deep vein thrombosis	13 (0.9)	117 (0.9)	0.77 (0.44–1.34)	0.3540
Cerebral vascular accident	67 (4.6)	447 (3.6)	1.13 (0.87–1.47)	0.3661
Acute myocardial infarction	22 (1.5)	75 (0.6)	2.06 (1.26–3.39)	0.0043*
Major adverse cardiac event^a^	89 (6.1)	531 (4.3)	1.24 (0.98–1.56)	0.0678
**Re-admission**				
In 90 days	252 (17.3)	1,291 (10.4)	1.52 (1.32–1.75)	<0.0001*
At the last follow up	814 (55.8)	5,507 (44.5)	1.39 (1.28–1.50)	<0.0001*
**Mortality due to any cause**				
In 90 days	15 (1.0)	37 (0.3)	2.55 (1.35–4.80)	0.0038*
At the last follow up	110 (7.5)	439 (3.5)	1.99 (1.59–2.48)	<0.0001*

PJI = periprosthetic joint infection; HR = hazard ratio; CI = confidence interval.

^a^ Any one of the cardiac outcomes.

^b^ Variables listed in Table 1 (except for follow-up years) were adjusted.

* *P* value < 0.05.

The CKD group had a higher re-admission rate due to any cause at both 3 months of follow-up (HR, 1.52; 95% CI, 1.33-1.75, *P*<0.0001) and at the end of follow-up (HR, 1.39; 95% CI, 1.28–1.50, *P*<0.0001). Compared to the non-CKD group, the CKD group also had a higher risk of mortality both at 3 months of follow-up (HR, 2.55; 95% CI, 1.35–4.80, *P*=0.0038) and at the last follow-up (HR, 1.99; 95% CI, 1.59–2.48, *P*<0.0001).

### Cumulative incidence of PJI

**Figure 3:**
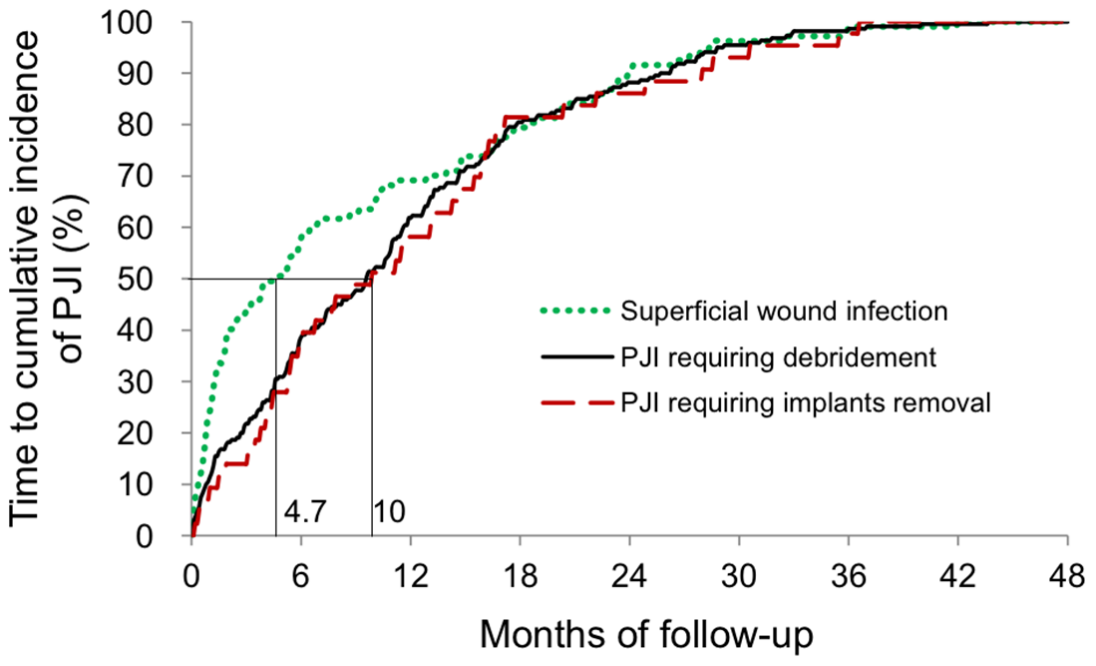
Time to the cumulative incidence of PJI Superficial wound infections developing earlier than PJIs requiring surgical procedures (median time, 4.7 months versus 10 months).

Forty-three (3.0%) and 283 (2.3%) patients suffered from TKA infections during follow-up in the CKD and non-CKD groups, respectively. Among these 326 patients, it took 4.7 months to reach 50% cumulative incidence of superficial wound infection (PJI just requiring antibiotic treatment), and approximately 10 months to reach 50% cumulative incidence of infection requiring simple surgical debridement or implant removal (Figure 3).

We used propensity score matching method to compare the difference between CKD cohort and Non-CKD cohort. We enrolled totally 2918 patients (1459 in each group). There was no significant difference in comorbidities between two groups (Supplementary Table 1) after matching. CKD group had a higher risk of urinary tract infection than control group after matching (Supplementary Table 2). In outcomes after discharge, CKD group had a comparable risk of AMI and MACE as control group after matching. For mortality, CKD group still have a higher risk of overall mortality and a borderline risk of mortality in 90 days after discharge than control group (*P*=0.0949, Supplementary Table 3).

## DISCUSSION

The main findings of this study are that the diabetic patients with CKD had a higher incidence of urinary tract infections in the index hospitalization for TKA compared to diabetic patients without CKD. In addition, the patients with CKD had higher risks of acute myocardial infarction, major adverse cardiac events, all-cause mortality, and readmission during follow-up compared to those without CKD. Furthermore, the patients with CKD had a trend of a higher risk of developing PJI than those without CKD. Therefore, orthopaedic surgeons should pay more attention to this group of patients.

The relationship between CKD and periprosthetic joint infections is controversial. CKD has been reported to an independent risk factor for PJI following elective hip and knee arthroplasty [5], and McCleery et al. [12] also reported that patients with CKD have a greater risk of developing early and late PJIs following TKA. In addition, Miric et al. [15] reported that patients with CKD have a higher incidence of superficial wound infections than those without CKD. Other studies have also shown that patients with CKD have a comparable risk of PJI to those without CKD regardless of the type of infection [16, 17]. However, most studies have not considered the influence of DM, which may play a pivotal role in the associated outcomes. In this study, the patients with CKD had a trend of developing PJIs following TKA compared to those without CKD after adjusting for confounding factors including DM.

Most superficial wound infections develop earlier than deep joint infections requiring surgical debridement or removal of the implants following index surgery regardless of the type of PJI. This may be due to the sequential treatment protocol, and because implant removal is always the ultimate procedure to control an infection. Further studies focusing on outcomes including duration of infection silence and survival with implants are needed to elucidate the efficacy of each treatment protocol on PJI.

Several articles have reported that impaired renal function is an independent risk factor for early 90-day mortality and 30-day morbidity after total joint arthroplasty [5, 9, 11, 17–20]. Ackland et al. [8] reported that patients with CKD had higher rates of pulmonary, infectious, and cardiovascular complications than those without CKD as well as a longer hospital stay by 4 days. In addition, Warth et al. [16] also demonstrated an increased risk of cardiac and systemic infectious complications as well as overall morbidity and mortality.

Mathew et al. [21] reported that patients with CKD had a higher incidence of postoperative death and cardiac vascular events than those without CKD during follow-up after non-cardiac surgery. Warth et al. [16] also reported that patients with CKD had a higher incidence of cardiac events, urinary tract infections and mortality than those without CKD after TKA, although the confounding factor of DM was not well evaluated since the CKD group had a significantly higher incidence of DM than the non-CKD group. The present study further demonstrates that patients with CKD have a higher risk of cardiac events and all-cause mortality after adjustments for all possible contributing confounding factors including DM.

### Strengths and limitations

There are three limitations to this study. First, although this study is a national health database study, it has it’s a natural retrospective design and relatively small number of patients. Second, we used ICD-9-CM codes to identify the diagnosis of CKD, and could not further divide the patients according to the severity of disease or clinical data. However, in Taiwan, the diagnosis of CKD is only attributed to a substantial deterioration in renal function, and therefore we can assume that these patients had at least stage III CKD. Further study to explore the associations between different severity of CKD and TKA outcome is required. Third, we used ICD-9-CM codes to identify the outcomes following TKA, and some minor adverse outcomes not requiring admission may have been missed. However, the critical identification of events ensured that the actual event rate could only have been more severe than our estimation, not lower.

There are also several strengths to this study. First, in contrast to previous studies, we stratified the effect of DM and further consolidated the effect of CKD itself on the surgical outcomes of TKA. Second, we not only used ICD-9-CM diagnostic codes to define PJI, but also validated the diagnoses using surgical codes in the NHIRD. This strict definition helped to strengthen the investigation of the relationship between the CKD and PJI.

In conclusion, compared to the diabetic patients without CKD, those with CKD had a higher incidence of urinary tract infection in the index admission, and higher risks of acute myocardial infarction, major adverse cardiac events, all-cause mortality, and readmission during follow-up. The patients with CKD also had a trend of a higher risk developing PJIs than those without CKD. To improve outcomes, orthopaedic surgeons should pay more attention to these patients.

## MATERIALS AND METHODS

### Data source

Data were obtained from the National Health Insurance Research Database (NHIRD) of Taiwan. This single-payer National Health Insurance (NHI) program was launched in 1995, and currently more than 23 million people (almost 99% of the population) are enrolled. The NHIRD contains claims data submitted by medical institutions through the NHI program. All clinical diagnoses are registered using International Classification of Diseases, 9th Revision, Clinical Modification [ICD-9-CM] codes (http://www.icd9-data.com/2007). The accuracy of the diagnoses of major diseases in the NHIRD such as DM or CKD has previously been validated [22–24]. All patient data are de-identified prior to analysis to ensure anonymity. This study was reviewed and approved by the Ethics Institutional Review Board of Chang Gung Memorial Hospital (IRB CGMH 103-5040B).

### Study cohort enrollment and exclusion criteria

All patients in the NHIRD with type 2 DM (ICD-9-CM code 250) were identified from January 1, 2008 to December 31, 2011 according to the method previously described [15]. Only patients with type 2 DM who were hospitalized for TKA between January 1, 2009 and June 30, 2011 were included in this study to ensure at least 1 year of previous medical records to identify comorbidities and exclusion conditions and to make sure each patient was followed for at least 6 months (Figure 1). The index hospitalization was defined as the admission for which the patient was admitted for TKA. CKD was defined according to the ICD-9-CM diagnostic codes detailed in Supplementary Table 1 [25]. The follow-up period was recorded from the index hospitalization to the date of death or until December 31, 2011.

In order to enroll patients with a first-ever procedure, the patients who had a history of TKA or total hip arthroplasty (THA) surgery were excluded. Patients with other factors that could confound the outcome were also excluded, including those with a history of osteomyelitis. Immunocompromised patients such as those undergoing dialysis were also excluded. Finally, 13844 patients were enrolled in the study cohort. These patients were then classified into two groups according to whether or not they had CKD. The study subject enrollment process is shown in Figure 1.

### Comorbidities and outcomes

Baseline comorbidities were identified by ICD-9-CM diagnostic codes before the index hospitalization. Chronic diseases such as DM and hypertension were defined as repeated diagnoses in separate clinic visits 1 year before the index admission. Acute comorbidities and events such as pulmonary embolism were defined as one inpatient primary diagnosis during a prior hospitalization.

In-hospital complications including PJI (i.e. superficial wound infection, surgical debridement, and implant removal), pneumonia, urinary tract infection, and death during the index admission were recorded. Infection outcomes were defined as composite outcomes of superficial wound infection, PJI requiring debridement, and PJI requiring implant removal during the follow-up period. A superficial wound infection was defined as readmission due to an infection only requiring antibiotic treatment. A PJI requiring debridement was defined as surgical debridement, arthrotomy, or capsulectomy requiring hospitalization. Cardiac outcomes included hospitalization due to pulmonary embolism, deep vein thrombosis, cerebral vascular event and acute myocardial infarction. A major adverse cardiac event was defined as any of the cardiac outcomes (Appendix 1). Deathwas identified by the method described in the previous study from the NHIRD in Taiwan [26].

### Statistical analysis

Baseline characteristics between the CKD and non-CKD groups were compared using the t-test for continuous variables and chi-square test for categorical variables. The incidence of in-hospital complications between the two groups was compared using multivariate logistic regression analysis with adjustments for all potential confounders listed in Table 1 except for follow-up years. With regards to the late complication after discharge of the index admission, we performed subdistribution hazard model in which death during the follow up was considered as a competing risk to compare group differences in which the aforementioned confounders were also adjusted for [27]. The cumulative incidence of long-term PJIs was estimated using the Kaplan-Meier method, and the log-rank test was used to compare group differences. All analyses were performed using SAS statistical software (version 9.4; SAS Institute Inc., Cary, NC). A *P* value < 0.05 was considered to be statistically significant.

## SUPPLEMENTARY MATERIALS TABLES



## Abbreviations

CI: confidence interval
CKD: chronic kidney disease
COPD: chronic obstructive pulmonary disease
DM: diabetes mellitus
HR: hazard ratio
ICD-9-CM: International Classification of Diseases, 9th Revision, Clinical Modification
NHI: National Health Insurance
NHIRD: National Health Insurance Research Database
OR: odds ratio
PJI: periprosthetic joint infection
SD: standard deviation
TKA: total knee arthroplasty
THA: total hip arthroplasty
