# Supplementary material for: Chronic kidney disease is associated with a risk of higher mortality following total knee arthroplasty in diabetic patients: a nationwide population-based study
Source: Oncotarget. 2017 Oct 31;8(59):100288–95. doi: 10.18632/oncotarget.22215 (PMC5725020; doi:10.18632/oncotarget.22215)
Supplement: Supplementary file 1 [file oncotarget-08-100288-s001.pdf]

Chronic kidney disease is associated with a risk of higher mortality following total knee arthroplasty in diabetic patients: a nationwide population-based study

SUPPLEMENTARY MATERIALS

Supplementary Table 1: Characteristics of the study cohort after propensity score matching

Variable	CKD (*n*=1458)	Non-CKD (*n*=1458)	*P* value
Age, Mean (*SD*)	71.6 (7.6)	71.6 (7.7)	0.9808
Age group			0.8925
< 65 years	241 (16.5)	232 (15.9)	
65-74 years	717 (49.2)	726 (49.8)	
≥ 75 years	500 (34.3)	500 (34.3)	
Gender			0.9370
Male	476 (32.6)	474 (32.5)	
Female	982 (67.4)	984 (67.5)	
Comorbidity			
Hypertension	1304 (89.4)	1330 (91.2)	0.1033
Dyslipidemia	611 (41.9)	623 (42.7)	0.6529
Gouty arthritis	365 (25.0)	360 (24.7)	0.8304
Cirrhosis	31 (2.1)	28 (1.9)	0.6932
Ischemic heart disease	420 (28.8)	425 (29.1)	0.8383
Rheumatoid arthritis	50 (3.4)	47 (3.2)	0.7567
COPD	128 (8.8)	131 (9.0)	0.8452
Immune disease	67 (4.6)	68 (4.7)	0.9298
Malignancy	99 (6.8)	78 (5.3)	0.1034
Pulmonary embolism	4 (0.3)	3 (0.2)	0.7050
Deep vein thrombosis	15 (1.0)	14 (1.0)	0.8520
Cerebral vascular accident	145 (9.9)	145 (9.9)	1.0000
Follow up (years)	1.9 (1.0)	2.0 (1.0)	0.0054*

CKD = chronic kidney disease; COPD = chronic obstructive pulmonary disease; SD = standard deviation.
* *P* value < 0.05.

Supplementary Table 2: In-hospital complications after propensity score matching

Variable	CKD (*n* = 1458)	Non-CKD (*n* = 1458)	CKD vs. Non-CKD OR (95% CI)	*P* value
Pneumonia	4 (0.3)	6 (0.4)	0.67 (0.19–2.36)	0.5292
Urinary tract infection	54 (3.7)	24 (1.6)	2.30 (1.41–3.74)	0.0008*
Acute PJI	5 (0.3)	6 (0.4)	0.83 (0.25–2.73)	0.7629
In hospital death	2 (0.1)	3 (0.2)	0.67 (0.11–3.99)	0.6566

PJI = periprosthetic joint infection; OR = odds ratio; CI = confidence interval.
* P value < 0.05.

Supplementary Table 3: Outcomes after discharge of the index admission after propensity score matching

Variables	CKD (*n* = 1458)	Non-CKD (*n* = 1458)	CKD vs. Non-CKD HR (95% CI)	*P* value
**Infection outcome**				
Superficial wound infection	14 (1.0)	11 (0.8)	1.28 (0.58–2.82)	0.5349
PJI requiring debridement	26 (1.8)	22 (1.5)	1.20 (0.68–2.12)	0.5199
PJI requiring implants removal	8 (0.5)	6 (0.4)	1.34 (0.47–3.87)	0.5856
Any PJI	43 (2.9)	35 (2.4)	1.25 (0.80–1.95)	0.3266
**Cardiac outcome**				
Pulmonary embolism	2 (0.1)	5 (0.3)	0.40 (0.08–2.06)	0.2723
Deep vein thrombosis	13 (0.9)	14 (1.0)	0.94 (0.44–1.98)	0.8605
Cerebral vascular accident	67 (4.6)	62 (4.3)	1.12 (0.79–1.57)	0.5355
Acute myocardial infarction	22 (1.5)	17 (1.2)	1.30 (0.69–2.45)	0.4126
Major adverse cardiac event[a]	89 (6.1)	78 (5.3)	1.17 (0.87–1.59)	0.3078
**Re-admission**				
In 90 days	252 (17.3)	170 (11.7)	1.55 (1.28–1.88)	<0.0001[*]
At the last follow up	813 (55.8)	668 (45.8)	1.40 (1.26–1.55)	<0.0001[*]
**Mortality due to any cause**				
In 90 days	15 (1.0)	7 (0.5)	2.15 (0.88–5.27)	0.0948
At the last follow up	110 (7.5)	72 (4.9)	1.60 (1.19–2.16)	0.0018[*]

PJI = periprosthetic joint infection; HR = hazard ratio; CI = confidence interval.
[a] Any one of the cardiac outcomes.
[*] P value < 0.05.

Supplementary 1. ICD-9-CM codes used for diagnosis in the current study

Variable	Code
Total knee arthroplasty	64164B<br>(Taiwan NHI procedure code)
Total hip arthroplasty	64162B<br>(Taiwan NHI procedure code)
Dialysis	585 (Catastrophic illness certificate)
Diabetes mellitus	250.xx
Chronic kidney disease	016.0, 042.xx, 095.4, 189.xx, 223.xx, 236.9, 250.4, 271.4, 274.1, 403.xx–404.xx, 440.1, 442.1, 446.21, 447.3, 572.4, 580.xx–589.xx, 590.xx–591.xx, 593.xx, 642.1, 646.2, 753.xx, and 984.xx
Hypertension	401.xx–405.xx
Dyslipidemia	272.xx
Gouty arthritis	274.xx
Cirrhosis	571.5, 571.6
Ischemic heart disease	410.xx–414.xx
Rheumatoid arthritis	714.xx
Chronic obstructive pulmonary disease	490.xx–496.xx
Immune diseases	710.0, 710.1, 714.0, 710.4, 710.3, 446.0, 446.2, 446.4, 446.5, 443.1, 446.7, 136.1, 694.4, 710.2, 555.xx, 556.xx
Malignancy	140.xx–208.xx
Pulmonary embolism	415.1x
Deep vein thrombosis	453.xx
Cerebral vascular accident	430.xx–437.xx
Pneumonia	480.xx–486.xx
Urinary tract infection	5990, 5950
Acute periprosthetic infection	730.xx, 99666, 99667, 99669, 99660
Superficial wound infection	99666, 99667, 99669, 99660
Debridement	48006C (Taiwan NHI procedure code)
Implant removal in the knee or hip	64198B (Taiwan NHI procedure code)
Acute myocardial infarction	410.xx
